# Association of systemic immune inflammatory index with all-cause and cause-specific mortality among individuals with type 2 diabetes

**DOI:** 10.1186/s12872-023-03638-5

**Published:** 2023-12-06

**Authors:** Chaoyang Chen, Yuwen Chen, Qiyue Gao, Qucheng Wei

**Affiliations:** 1 Department of Cardiology, Shangyu People’s Hospital of Shaoxing, Shaoxing, China; 2 Department of Cardiology, Second Affiliated Hospital, Zhejiang University School of Medicine, Hangzhou, China

**Keywords:** Diabetes, Inflammation, Mortality, Risk factor, Systemic immune inflammatory index

## Abstract

**Background:**

The evidence regarding the association between the systemic immune inflammatory index (SII) and mortality among individuals with diabetes is limited. This study aims to evaluate the associations between SII and all-cause and cause-specific mortality among individuals with diabetes.

**Methods:**

The study included 8,668 participants with diabetes from the National Health and Nutrition Examination Survey (NHANES) 1999–2018 with follow-up until 31 December 2019. The calculation of SII in this study was performed using the following formula: the neutrophil-to-lymphocyte ratio multiplied by the platelet count (10^9 cells/µL).

**Results:**

The study documented 2,463 deaths over 68,542 person-years, including 853 deaths from CVD and 424 from cancer. An increase in SII was significantly associated with higher all-cause and CVD mortality risk after multivariate adjustment. For each standard deviation increment in natural log transformed SII (lnSII), all-cause mortality increased by 17%, and CVD mortality increased by 34% (both *P* < 0.001). Additionally, the association between SII and all-cause mortality was U-shaped, with the inflection point at 6.02. The association between SII and CVD mortality was non-linear and J-shaped, where the risk increased significantly when lnSII exceeded 6.22. Furthermore, the association between SII and CVD mortality was attenuated in female and hyperlipidemia patients.

**Conclusion:**

In this study, we observed a significant positive association between the SII and both all-cause and CVD mortality in patients with diabetes. Additionally, it was discovered that this association exhibited a non-linear pattern. These findings suggest that maintaining SII within an optimal range may play a critical role in mitigating the risk of mortality.

**Supplementary Information:**

The online version contains supplementary material available at 10.1186/s12872-023-03638-5.

## Introduction

Diabetes is a major public health issue that has a significant impact on both human life and healthcare expenditures. Type 2 diabetes and its complications are major contributors to mortality and disability worldwide [1–3]. Cardiovascular disease (CVD), including coronary heart disease, peripheral vascular disease, and cerebrovascular disease, is the leading cause of morbidity and mortality in the United States [4]. CVD typically develops earlier in individuals with type 2 diabetes [5], highlighting the need to identify potential risk factors for screening and intervention.

Inflammation has been identified as a critical factor in the development and progression of atherosclerosis [6, 7]. As a result, targeting inflammation for the primary and secondary prevention of CVD has been a major area of research focus [8–10]. The systemic immune-inflammation index (SII) is a novel inflammatory marker that can comprehensively reflect the degree of inflammation and immune status. SII can be easily calculated using the information from complete blood count, which is a routine laboratory test in medical practice. Although initially proposed as a predictive marker for cancer [11], recent studies have demonstrated that SII was associated with the prognosis of individuals among the general population, CVD, or hypertension [12–15]. However, the association between SII and all-cause and cause-specific mortality has not been well studied among individuals with type 2 diabetes. Assess the association between SII and long-term mortality risk among individuals with diabetes holds pivotal importance, as it facilitates a profound comprehension of the influence of inflammation and immune status on the health status of diabetic patients. Moreover, such investigation offers valuable insights for enhancing clinical management and intervention strategies for patients.

Therefore, we conducted a prospective study to assess the association between SII and all-cause and cause-specific mortality in a nationwide representative cohort of United States participants with type 2 diabetes.

## Method

### Study population

The National Health and Nutrition Examination Survey (NHANES) is a study conducted nationwide in the United States, with the goal of collecting data on the nutrition and health of noninstitutionalized civilians. To obtain baseline data, various information such as demographic details, physical examinations, and laboratory tests were gathered at both the participants’ homes and a mobile center. The protocol used for this study was approved by the Institutional Review Board of the National Center for Health Statistics, and all participants were required to provide informed consent when enrolling in the survey.

**Fig. 1:**
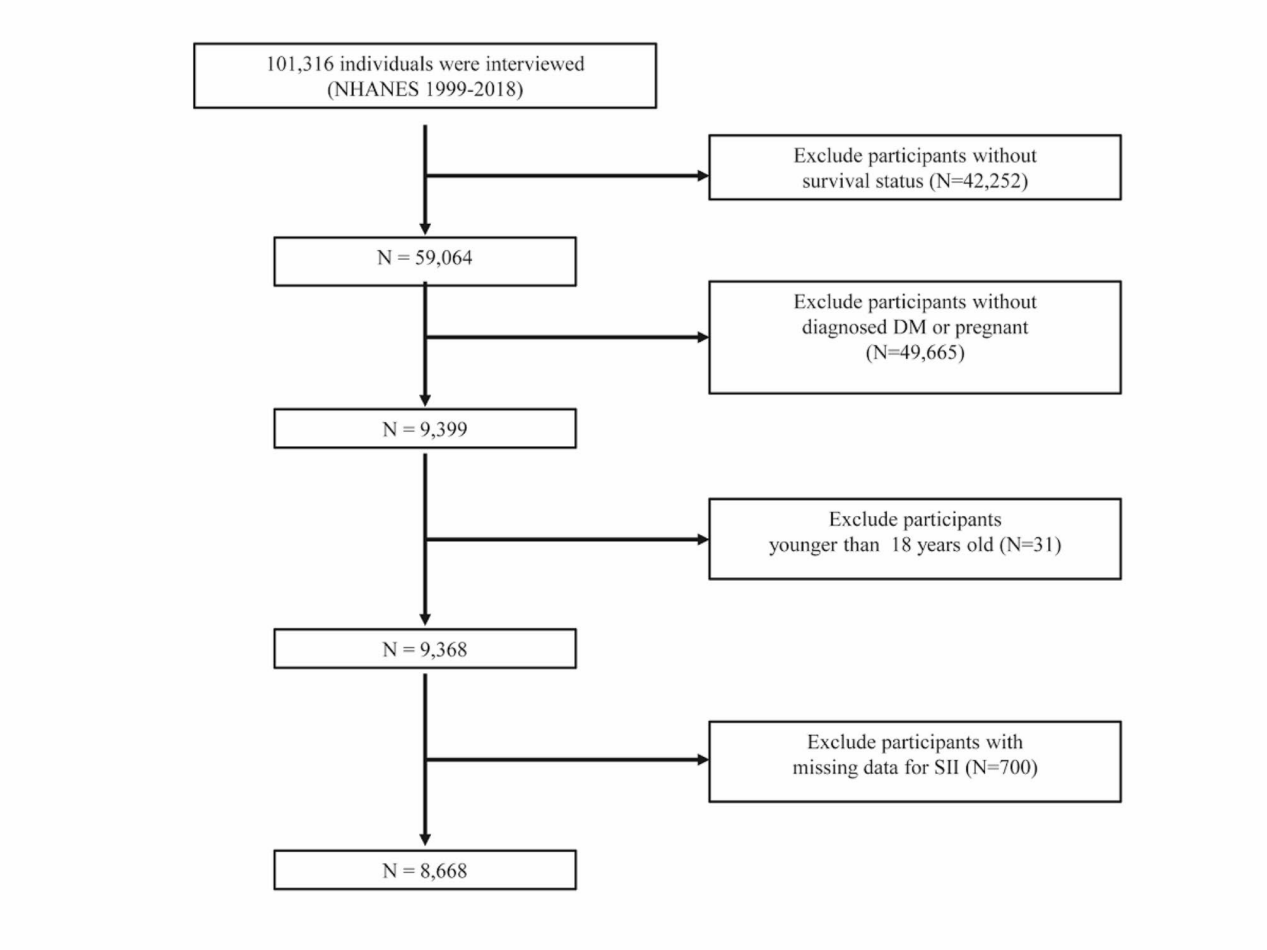
Flow diagram of the selection of eligible individuals

For this study, we utilized data from ten NHANES cycles conducted between 1999 and 2018, encompassing a total of 101,316 individuals. We excluded subjects who lacked survival status (n = 42,252), pregnant individuals, and those without a diagnosis of type 2 diabetes (n = 49,665). Additionally, individuals below 18 years old (n = 31) and those with missing SII data (n = 700) were also excluded. Following the application of these exclusion criteria, a total of 8,668 subjects with type 2 diabetes were included in this study. Type 2 diabetes was defined based on one of the following criteria: (1) self-reported doctor-diagnosed diabetes; (2) fasting blood glucose ≥ 7.0 mmol/L; (3) two-hour oral glucose tolerance test blood glucose ≥ 11.1 mmol/L; (4) glycated hemoglobin A1c (HbA1c) ≥ 6.5%; or (5) use of hypoglycemic medication. Figure 1 provides a visual representation of the detailed participant selection process.

### Definition of SII

The calculation of the SII in this study involved multiplying the neutrophil-to-lymphocyte ratio by the platelet count (10^9 cells/µL), as described in a previous investigation [11]. As the distribution of SII exhibited a right-skewed pattern, the variable was assessed in its continuous form after applying a natural log transformation (lnSII). Subsequently, the lnSII variable was divided into four equal subgroups.

### Assessment of mortality

To determine all-cause, CVD, and cancer mortality rates, the study participants were linked to the National Death Index until December 31, 2019. Cause-specific deaths were identified using the International Classification of Diseases, Tenth Revision (ICD-10) codes. CVD mortality was defined by ICD-10 codes I00-I09, I11, I13, I20-I51, or I60-I69, while cancer mortality was defined by ICD-10 codes C00-C97.

### Covariates assessment

Age, gender, ethnicity, education, family income, smoking and drinking habits, comorbidity disease status, and diabetes medication use were assessed through structured interviews conducted at the participants’ homes. At the mobile center, measurements of body mass index (BMI) and collection of blood samples were performed. Ethnicity was classified into four categories: Mexican American, non-Hispanic White, non-Hispanic Black, or other. Education level was categorized as less than high school, high school or equivalent, or college or higher. The ratio of family income to poverty was classified into three groups: 0–1.0, 1.0–3.0, or > 3.0. Smoking status was defined as either a never smoker (smoked < 100 cigarettes in their lifetime), current smoker (smoked ≥ 100 cigarettes in their lifetime and currently smokes some days or every day), or former smoker (smoked ≥ 100 cigarettes in their lifetime and currently does not smoke). Drinking status was grouped as nondrinker (< 12 drinks in their lifetime), low-to-moderate drinker (≤ 1 drink per day for females, ≤ 2 drinks per day for males, or binge drinking on < 2 days per month), heavy drinker (> 1 drink per day for females, > 2 drinks per day for males, or binge drinking on ≥ 2 days per month), or former drinker (≥ 12 drinks in their lifetime and did not drink last year).

Hypertension was defined based on meeting one of the following criteria: (1) self-reported doctor-diagnosed hypertension; (2) mean systolic blood pressure ≥ 140 mmHg or mean diastolic blood pressure ≥ 90 mmHg; or (3) use of antihypertensive medication. Hyperlipidemia was defined based on meeting one of the following criteria: (1) self-reported doctor-diagnosed hyperlipidemia; (2) triglyceride (TG) levels ≥ 150 mg/dL, total cholesterol (TC) levels ≥ 200 mg/dL, high-density lipoprotein (HDL) levels < 40 mg/dL, low-density lipoprotein (LDL) levels ≥ 130 mg/dL; or (3) use of antihyperlipidemic medication. Atherosclerotic cardiovascular disease (ASCVD) was defined as the presence of coronary heart disease, heart attack, angina, or stroke.

At the time of recruitment, various laboratory measurements were conducted, including complete blood count, plasma glucose, HbA1c, TG, TC, HDL, and LDL. To assess insulin resistance, the homeostatic model assessment of insulin resistance (HOMA-IR) was calculated using a previously established method [16]. Further information can be found on the official website (http://www.cdc.gov/nchs/nhanes/).

### Statistical analysis

To account for the complex sampling design of NHANES, all analyses incorporated sample weights, clustering, and stratification. Person-time was calculated from the recruitment date until either the date of death or the end of follow-up (December 31, 2019), whichever occurred first. Weighted means ± standard error (SE) was used for continuous variables, while frequency and weighted percentages were used for categorical variables.

The associations between lnSII and all-cause and cause-specific mortality were estimated using multivariate Cox regression models. Model 1 adjusted for age, sex, and ethnicity. Model 2 further adjusted for BMI, education level, family income-poverty ratio, smoking status, and drinking status. Model 3 additionally adjusted for duration of diabetes, diabetes medication use, HbA1c, and the presence of hypertension, hyperlipidemia, ASCVD, and chronic kidney disease (CKD). The linear trend was assessed by treating the median value of each category as a continuous variable. Multiple imputation was employed to handle missing values for variables.

To assess the relationship between lnSII and all-cause and cause-specific mortality, restricted cubic spline regression with four knots was employed. This analysis incorporated the multivariate adjustment. In order to evaluate potential nonlinearity, a likelihood ratio test was performed. If nonlinearity was detected, a two-piece Cox proportional hazards regression model was utilized, which allows for nonlinearity at the inflection point. The inflection point represents the point at which the relationship between the predictor and outcome variables undergoes a change and was used to determine the two separate components of the model.

Stratified analyses were conducted, considering factors such as age, gender, ethnicity, BMI, smoking status, HbA1c, duration of diabetes, hypertension, and hyperlipidemia. The significance of interactions between lnSII and stratification variables was evaluated using the P value for the product terms.

Sensitivity analyses were performed to test the robustness of the results. Exclusion criteria included individuals with a history of ASCVD or cancer, as well as those who died within 2 years of follow-up to minimize reverse causation bias. Additionally, the multivariate model was further adjusted to include the healthy eating index (HEI) for dietary factors, as well as HOMA-IR, TG, HDL, and LDL. Statistical significance was defined as a two-sided P value < 0.05. All analyses were conducted using R version 4.2.3.

## Results

### Baseline characteristics

A total of 8,668 individuals with type 2 diabetes were included in the study, with a mean age of 59.19 (0.24) years. Of these, 4,459 (51.44%) were male and 4,209 (48.56%) were female. The weighted mean (95% CI) level of lnSII was 6.24 (0.01). Table 1 presents the baseline characteristics of the study population according to lnSII levels. Individuals in the highest lnSII quartile were more likely to be female, non-Hispanic White, obese, and comorbid with CKD. They also had lower HEI levels and higher education levels. Supplementary Table 1 presents baseline characteristics based on all-cause mortality.

**Table 1:** Baseline Characteristics of Participants with Diabetes according to lnSII

	lnSII				
	Total	≤ 5.84	5.84–6.19	6.19–6.55	> 6.55
Participants, No.	8668	2165	2168	2170	2165
Age, mean (SE), y	59.19(0.24)	59.35(0.43)	59.05(0.41)	59.26(0.41)	59.13(0.47)
Gender					
Male	4459(51.44)	1196(54.60)	1101(51.87)	1103(50.80)	1059(47.19)
Female	4209(48.56)	969(45.40)	1067(48.13)	1067(49.20)	1106(52.81)
Ethnicity					
Non-Hispanic White	3175(36.63)	594(52.75)	703(59.70)	886(65.48)	992(69.47)
Non-Hispanic Black	2113(24.38)	763(22.98)	512(13.53)	445(11.58)	393(10.16)
Mexican American	1759(20.29)	396( 9.53)	497(10.96)	455( 8.81)	411( 7.79)
Other	1621(18.7)	412(14.73)	456(15.81)	384(14.14)	369(12.59)
BMI					
<25.0	1200(14.27)	292(13.71)	298(11.72)	286(10.70)	324(13.88)
25.0-29.9	2554(30.37)	707(30.92)	665(27.13)	618(27.96)	564(24.63)
≥30	4655(55.36)	1118(55.37)	1153(61.15)	1195(61.34)	1189(61.50)
HEI, mean (SE)	51.16(0.22)	52.41(0.44)	51.25(0.41)	51.19(0.41)	50.05(0.42)
Smoking status					
Never	4320(49.97)	1141(51.61)	1107(49.87)	1072(49.32)	1000(47.21)
Current	1406(16.26)	337(16.78)	331(15.90)	355(16.62)	383(17.29)
Former	2920(33.77)	680(31.62)	725(34.23)	739(34.06)	776(35.51)
Drinking status					
Never	1458(18.91)	355(16.65)	378(16.72)	385(17.32)	340(14.42)
Mild-to-moderate	2374(30.79)	589(33.59)	579(35.13)	611(34.69)	595(36.35)
Heavy	1708(22.15)	423(24.57)	449(25.50)	443(24.37)	393(22.62)
Former	2170(28.15)	553(25.19)	517(22.65)	512(23.62)	588(26.61)
Education levels					
Less than high school	3231(37.34)	859(29.56)	840(26.31)	771(22.56)	761(23.96)
High school or equivalent	1976(22.84)	480(25.17)	500(26.39)	484(24.79)	512(25.98)
College or above	3445(39.82)	821(45.27)	827(47.31)	911(52.64)	886(50.06)
Family income-poverty ratio					
≤ 1.0	1868(23.9)	479(18.36)	462(16.73)	455(15.69)	472(16.74)
1.0–3.0	3631(46.45)	916(43.79)	871(40.51)	919(40.62)	925(42.84)
>3.0	2318(29.65)	549(37.85)	617(42.76)	610(43.70)	542(40.41)
Duration of diabetes					
≤3 years	4179(48.21)	1099(52.14)	1036(50.52)	1065(49.54)	979(48.76)
3–10 years	1923(22.19)	485(23.74)	515(23.05)	446(21.32)	477(21.37)
>10 years	2566(29.6)	581(24.12)	617(26.43)	659(29.14)	709(29.87)
FBG, mmol/L	8.43(0.06)	8.34(0.14)	8.60(0.14)	8.33(0.12)	8.45(0.11)
HOMA-IR	8.64(0.23)	8.64(0.42)	8.31(0.45)	8.66(0.37)	8.90(0.50)
HbA1c, %					
<7.0	4906(56.76)	1234(59.27)	1197(57.73)	1222(60.18)	1253(59.81)
≥7.0	3737(43.24)	925(40.73)	964(42.27)	939(39.82)	909(40.19)
Diabetes medication use					
No insulin or pills	3266(37.68)	865(42.35)	798(38.97)	845(39.20)	758(36.56)
Only diabetes pills	3741(43.16)	935(40.39)	983(44.64)	920(42.78)	903(39.68)
Only insulin	799(9.22)	168( 8.62)	172( 7.02)	189( 8.35)	270(13.56)
Pills and insulin	862(9.94)	197( 8.64)	215( 9.37)	216( 9.67)	234(10.20)
Self-reported disease					
Hypertension	6208(71.64)	1539(68.25)	1533(68.12)	1550(70.41)	1586(70.29)
Hyperlipidemia	7479(86.29)	1828(86.57)	1883(88.30)	1913(89.05)	1855(86.65)
ASCVD	2012(23.27)	479(22.47)	442(20.20)	528(22.92)	563(22.33)
CKD	3503(41.74)	767(32.58)	798(34.31)	902(39.38)	1036(42.08)
TG, mmol/L	1.93(0.04)	2.00(0.08)	1.98(0.08)	1.94(0.05)	1.81(0.06)
TC, mmol/L	4.89(0.02)	4.99(0.04)	4.91(0.03)	4.91(0.04)	4.79(0.03)
HDL, mmol/L	1.23(0.01)	1.24(0.01)	1.23(0.01)	1.22(0.01)	1.25(0.01)
LDL, mmol/L	2.77(0.02)	2.81(0.04)	2.85(0.04)	2.73(0.04)	2.71(0.04)

**Notes**: Data are numbers (percentages) unless otherwise indicated. All estimates accounted for complex survey designs, and all percentages were weighted. **Abbreviationss**: ASCVD, Atherosclerotic cardiovascular disease; BMI, Body mass index; CKD, Chronic kidney disease; FBG, Fasting blood glucose; HDL, High-density lipoprotein; HEI, Healthy Eating Index; HbA1c, Glycated hemoglobin A1c; HOMA-IR, Homeostatic model assessment of insulin resistance; LDL, Low-density lipoprotein; NHANES, National Health and Nutrition Examination Survey; SII, Systemic immune inflammatory index; TC, Total cholesterol; TG, Total triglyceride.

### SII and mortality

During 68,542 person-years of follow-up, a total of 2,463 deaths were documented, including 853 deaths due to CVD and 424 deaths due to cancer. Univariate analysis of the Cox regression model is presented in Supplementary Table 2. As shown in Table 2, after adjusting for age, gender, ethnicity, BMI, smoking and drinking status, diabetes duration, diabetes medication use, and comorbidities, each one-standard deviation increased in lnSII was associated with a 17% increased risk of all-cause mortality and a 34% increased risk of CVD mortality (both *P* < 0.001). However, no significant association was found between SII and cancer mortality. Furthermore, higher quartiles of lnSII were significantly associated with a higher risk of all-cause and CVD mortality when compared to the lowest quartile. The multivariate-adjusted HRs and 95% CIs from lowest to highest lnSII categories (≤ 5.84, 5.84–6.19, 6.19–6.55, > 6.55) were 1.00 (reference), 0.95 (0.79, 1.16), 0.92 (0.75, 1.12), 1.33 (1.10, 1.61), respectively, for all-cause mortality (Ptrend = 0.002); 1.00 (reference), 1.03 (0.79, 1.34), 1.07 (0.77, 1.50), 1.59 (1.11, 2.27), respectively, for CVD mortality (Ptrend = 0.01); and 1.00 (reference), 0.61 (0.38, 0.96), 0.59 (0.38, 0.91), 0.95 (0.62, 1.44), respectively, for cancer mortality (Ptrend = 0.96).

**Table 2:** Multivariable Cox Regression Analyses for Mortality among Participants with Diabetes

	lnSII					Per SD increment in lnSII
	≤ 5.84	5.84–6.19	6.19–6.55	> 6.55	*P*_trend_	
All-cause mortality						
Death, No./total No.	510/2165	547/2168	614/2170	792/2165		
Model 1	Reference	0.99(0.85,1.16)	0.97(0.81,1.15)	1.44(1.25,1.65)	< 0.001	1.21(1.14,1.28)
Model 2	Reference	0.94(0.79,1.13)	0.97(0.79,1.18)	1.42(1.19,1.68)	< 0.001	1.21(1.12,1.30)
Model 3	Reference	0.95(0.79,1.16)	0.92(0.75,1.12)	1.33(1.10,1.61)	0.002	1.17(1.07,1.26)
CVD mortality						
Death, No.	178	191	220	264		
Model 1	Reference	1.10(0.89,1.37)	1.03(0.77,1.37)	1.65(1.25,2.18)	< 0.001	1.33(1.19,1.50)
Model 2	Reference	0.96(0.75,1.24)	1.05(0.77,1.43)	1.63(1.19,2.25)	0.002	1.38(1.20,1.58)
Model 3	Reference	1.03(0.79,1.34)	1.07(0.77,1.50)	1.59(1.11,2.27)	0.01	1.34(1.15,1.57)
Cancer mortality						
Death, No.	100	97	93	134		
Model 1	Reference	0.77(0.52,1.15)	0.67(0.45,0.99)	1.09(0.77,1.54)	0.69	1.07(0.90,1.28)
Model 2	Reference	0.65(0.41,1.01)	0.60(0.39,0.93)	1.02(0.68,1.53)	0.81	1.04(0.84,1.28)
Model 3	Reference	0.61(0.38,0.96)	0.59(0.38,0.91)	0.95(0.62,1.44)	0.96	0.99(0.80,1.23)

**Notes**: Model 1: adjusted for age (continuous), sex (male or female) and ethnicity (non-Hispanic white, non-Hispanic black, Mexican American, or other); Model 2: further adjusted for BMI (< 25, 25–30, ≥ 30 kg/m^2^), education level (less than high school, high school or equivalent, or college or above), family income-poverty ratio (0–1.0, 1.0–3.0, or > 3.0), smoking status (never smoker, current smoker, or former smoker), drinking status (non-drinker, low-to-moderate drinker, heavy drinker, or former drinker); Model 3: further adjusted for duration of diabetes (≤ 3, 3–10, or > 10 years), diabetic medication use (none, only oral medication, insulin, or others), HbA1c (< 7%, or ≥ 7%), hypertension, hyperlipidemia, ASCVD, CKD (yes, or no). **Abbreviationss**: CVD, cardiovascular disease; SII, systemic immune inflammation

### Dose-response relationship between SII and mortality

**Fig. 2:**
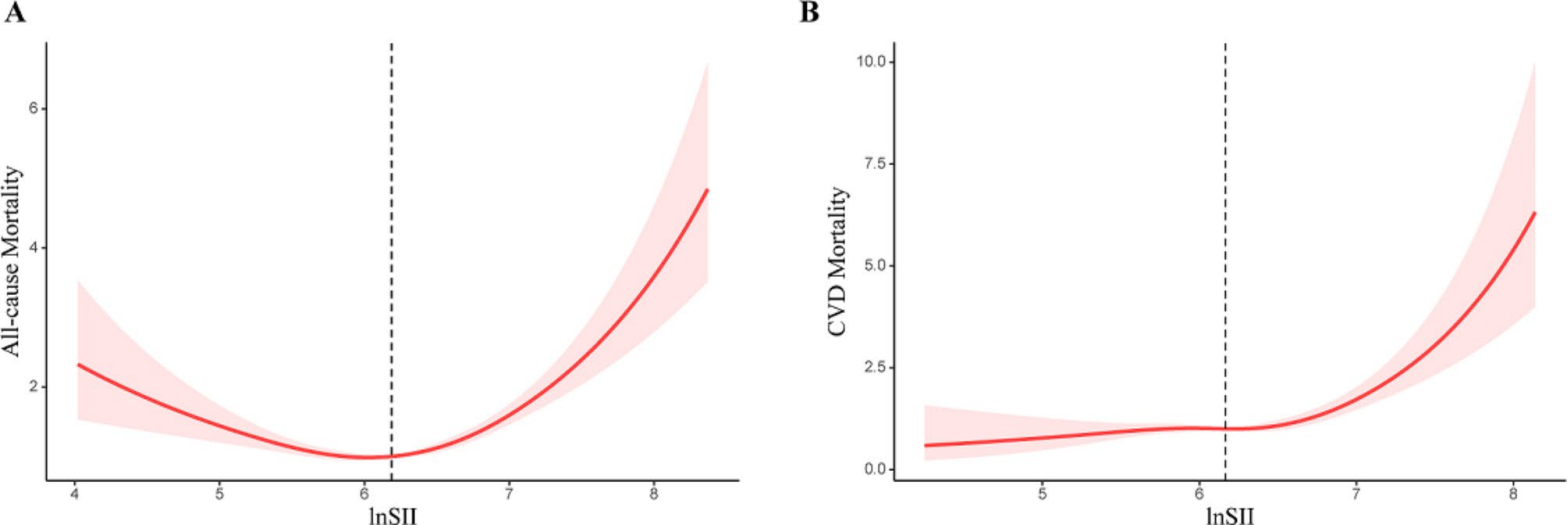
Restricted cubic spline regression for the associations between lnSII and all-cause mortality (A) and CVD mortality (B)

After adjusting for age, gender, ethnicity, BMI, smoking and drinking status, diabetes duration, diabetes medication use, and comorbidities, a nonlinear and U-shaped association was observed between lnSII and all-cause mortality, and a J-shaped association was observed between lnSII and CVD mortality (P nonlinearity < 0.05) (Fig. 2). As shown in Table 3, two-piece Cox proportional hazards regression model was used and the risk of all-cause mortality decreased to the minimum at point lnSII 6.03 (HR: 0.64, 95% CI 0.48–0.86) and then increased with elevated lnSII (HR: 1.92, 95% CI 1.65–2.25). For CVD mortality, when lnSII exceeded 6.22, the risk increased significantly (HR:2.55, 95% CI 1.69–3.84). A U-shaped association was observed between lnSII and cancer mortality (Supplementary Fig. 1), and the two-piecewise linear regression suggested that the risk of cancer mortality reached a minimum at lnSII of 6.07 (HR: 0.30, 95% CI 0.17–0.53) and then increased (HR: 2.51, 95% CI 1.75–3.61).

**Table 3:** Nonlinearity Addressed through Two-piecewise Linear Model

	All-cause mortality	*P*	CVD mortality	*P*	Cancer mortality	*P*
Threshold value	6.03		6.22		6.07	
< Threshold value	0.64(0.48,0.86)	0.003	1.07(0.68,1.69)	0.76	0.30(0.17,0.53)	< 0.001
> Threshold value	1.92(1.65,2.25)	< 0.001	2.55(1.69,3.84)	< 0.001	2.51(1.75,3.61)	< 0.001

**Notes**: hazard ratios were calculated based on multivariable Cox proportional hazards regression model adjusted for age (continuous), sex (male or female) and ethnicity (non-Hispanic white, non-Hispanic black, Mexican American, or other), BMI (< 25, 25–30, ≥ 30 kg/m2), education level (less than high school, high school or equivalent, or college or above), family income-poverty ratio (0–1.0, 1.0–3.0, or > 3.0), smoking status (never smoker, current smoker, or former smoker), drinking status (non-drinker, low-to-moderate drinker, heavy drinker, or former drinker), duration of diabetes (≤ 3, 3–10, or > 10 years), diabetic medication use (none, only oral medication, insulin, or others), HbA1c (< 7%, or ≥ 7%), hypertension, hyperlipidemia, ASCVD, CKD (yes, or no). **Abbreviations**: CVD, cardiovascular disease

### Subgroup analyses and sensitivity analyses

**Fig. 3:**
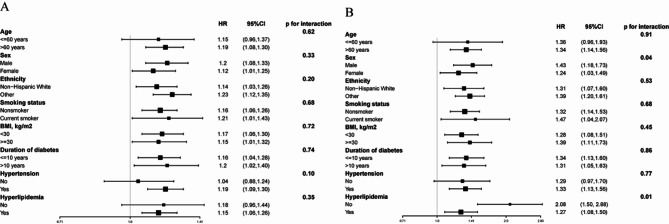
Forest plot for subgroup analysis of associations between lnSII and all-cause and CVD mortality. Hazard ratios (HR) were calculated using multivariate COX proportional hazards models adjusted for variables in model 3 except for the variable used for stratification

Subgroup analyses stratified by age, gender, ethnicity, BMI, smoking status, duration of diabetes, and comorbidities showed consistent results with the overall analysis (Fig. 3 and Supplementary Tables 3–4). No significant interactions were detected between lnSII and these stratifying variables, except for gender and hyperlipidemia for CVD mortality (*P* < 0.05). In female participants with hyperlipidemia, the association of lnSII with CVD mortality was attenuated. However, as shown in Supplementary Fig. 2, the results of RCS stratified by gender and hyperlipidemia demonstrated that there were still J-shaped associations between lnSII and CVD mortality in both male and female, non-hyperlipidemia, and hyperlipidemia subgroups.

In the sensitivity analyses, excluding individuals with a history of ASCVD or cancer did not significantly alter the results (Supplementary Tables 5–6). The results remained unchanged when participants who died within the first 2 years of follow-up were excluded (Supplementary Table 7). Similarly, after further adjusting for HEI, HOMA-IR, TG, HDL, and LDL, the results were consistent (Supplementary Table 8). Supplementary Table 9 provides the proportion of missing data, while Supplementary Fig. 3 demonstrates the consistency of the results with multiple imputations.

## Discussion

This prospective cohort study of individuals with type 2 diabetes found that higher levels of SII were significantly associated with higher CVD mortality. The association of lnSII with CVD mortality was J-shaped, with a significant increase in risk when lnSII exceeded 6.22. Additionally, there were U-shaped associations between lnSII and all-cause and cancer mortality and the inflection points of lnSII with the lowest HR were 6.03 and 6.07, respectively. These associations were independent of traditional risk factors, including lifestyle factors, BMI, duration of diabetes, diabetes medication use, and comorbidities. Stratified analyses and sensitivity analyses supported the robustness of these findings. As far as we know, this study is the first to assess the association of SII with all-cause and cause-specific mortality among individuals with type 2 diabetes.

Inflammation plays a significant role in the development and progression of diabetes. Proinflammatory cytokine TNFα can activate intracellular inflammatory signaling, leading to insulin resistance and glucose intolerance [17, 18]. Additionally, microvascular occlusion, common in diabetes, can cause hypoxia and trigger an inflammatory response [19]. Atherosclerosis is also an inflammatory process [20], which is not only a complication of metabolic dysfunction in diabetes but also a result of metabolic stress-induced inflammation [21]. Chronic inflammation has been shown to play a critical role in the pathogenesis of CVD [22, 23]. While many anti-diabetic drugs are available, optimal glucose control alone is often insufficient to prevent the long-term complications of diabetes [21]. Studies have demonstrated that anti-inflammatory therapy in diabetes could improve glycemia and increase insulin secretion [24–26].

SII is a composite variable that combines platelet and neutrophil-to-lymphocyte ratio and can comprehensively assess the degree of inflammation and immune status. Previous studies have indicated that SII is associated with the complications of diabetes [27, 28]. Zhen et al. included 13 studies with 152,996 participants and found that SII was significantly associated with higher risk of CVD in stroke, myocardial infarction, and peripheral arterial disease patients [29]. Similarly, Xia et al. found similar results [12]. Platelet hyperactivity has a principal role in the pathophysiology of atherogenesis [30], and in participants with diabetes, the adherence of platelets to the endothelium and their aggregation are more frequent compared to healthy individuals [31]. Additionally, neutrophil-to-lymphocyte ratio also increases the risk of CVD among individuals with diabetes [32]. In patients afflicted with CVD, there appears to be a significant association between SII and prognostic outcomes. As observed in the study conducted by Xiao et al., the relationship between SII and various mortality causes - namely, all-cause mortality, CVD-specific mortality, and tumor-related mortality - in CVD patients exhibited a U-shaped pattern [33]. This suggests that both low and high SII levels may be associated with increased mortality risk. Similarly, research led by Huang et al. revealed a notable positive correlation between SII and both in-hospital mortality and long-term adverse prognostic outcomes, specifically within the demographic of older myocardial infarction patients [34].

Our findings partially support those of previous studies. In our study, we made a notable observation regarding the relationship between SII and all-cause mortality as well as cardiovascular mortality in diabetes patients, after meticulous adjustment for multiple confounding factors. Specifically, we found a U-shaped association of lnSII, used as a composite index, with the risk of all-cause and cancer mortality. This was making sense as low platelets usually means increased risk of bleeding and disseminated intravascular coagulation in cancer which may contribute to all-cause and cancer mortality. However, in contrast to hypertensive individuals [13], our study found a nonlinear, J-shaped association between lnSII and CVD mortality in individuals with diabetes, indicating that when lnSII exceeded the inflection point, the influence of inflammation on CVD became more deleterious.

The current study found that the association between lnSII and CVD mortality was attenuated in females, which may be due to the protective effect of female hormones that reduce the degree of inflammation and prevent atherosclerosis. This finding is consistent with previous studies that have shown that premenopausal women have a lower risk of CVD compared to men, and this difference is partly attributed to the protective effect of estrogen [35]. Additionally, the study found that the influence of inflammation on CVD was attenuated in patients with hyperlipidemia. This finding is supported by the independent association of hyperlipidemia with endothelial dysfunction, platelet activation, and aggregation, which overlap with the pathophysiological mechanisms of inflammation in CVD [36]. However, the exact mechanisms underlying this interaction between hyperlipidemia and inflammation in CVD require further investigation.

The implications of this study propose that the SII could serve as a potential prognostic marker for adverse outcomes in patients with diabetes. The SII, within the context of patients with diabetes, can serve as a barometer for the level of systemic inflammation. Modulating the SII within a defined range may potentially enhance the prognosis for these patients. Further studies are needed to explore the effect of these indices, such as triglyceride-glucose index [37], and using AI based systems to provide precise predictions [38].

The current study has several strengths, including its relatively large sample size and consideration of many potential confounding factors. Additionally, the study was based on a nationwide representative cohort of individuals with type 2 diabetes, which enhances the generalizability of the findings. However, several limitations should also be considered. Firstly, due to the observational study design, the findings of the study cannot establish a causality between SII and mortality outcomes. Secondly, the SII levels were analyzed based on a single serum measurement, which may not accurately reflect the long-term status of immune inflammation. Thirdly, the covariates collected at baseline may change over time, potentially attenuating the true association between SII and all-cause and cause-specific mortality. Fourthly, the severity of diabetes could not be assessed sufficiently due to lack of necessary information. Finally, residual or unknown confounding factors cannot be excluded.

## Conclusion

The current study found a significant association between higher SII levels and increased CVD mortality among individuals with diabetes. The J-shaped association between lnSII and CVD mortality, with an extremely increased risk when lnSII exceeded 6.22, suggest that an optimal range of SII may be crucial for reducing the risk of mortality in this population. Additionally, the U-shaped associations between lnSII and all-cause and cancer mortality, with inflection points of 6.03 and 6.07, respectively, suggests that both low and high levels of SII may be associated with increased mortality risk in individuals with type 2 diabetes. Identifying and treating chronic inflammation may be a promising strategy to reduce the risk of mortality in individuals with type 2 diabetes.

### Electronic supplementary material

Below is the link to the electronic supplementary material.

Supplementary Material 1

Supplementary Material 2

Supplementary Material 3

Supplementary Material 4

Supplementary Material 5

Supplementary Material 6

Supplementary Material 7

Supplementary Material 8

Supplementary Material 9

Supplementary Material 10

Supplementary Material 11

Supplementary Material 12

Supplementary Material 13

## Data Availability

We utilized the NHANES database in our study, and the details can be accessed at: http://www.cdc.gov/nchs/nhanes/.

## List of abbreviations

ASCVD: Atherosclerotic cardiovascular disease
BMI: Body mass index
CKD: Chronic kidney disease
CVD: Cardiovascular disease
HDL: High-density lipoprotein
HEI: Healthy eating index
HOMA-IR: Homeostatic model assessment of insulin resistance
LDL: Low-density lipoprotein
NHANES: National Health and Nutrition Examination Survey
SII: Systemic immune inflammatory index
TC: Total cholesterol
TG: Triglyceride
