# Supplementary material for: Association of systemic immune inflammatory index with all-cause and cause-specific mortality among individuals with type 2 diabetes
Source: BMC Cardiovasc Disord. 2023 Dec 6;23:596. doi: 10.1186/s12872-023-03638-5 (PMC10702126; doi:10.1186/s12872-023-03638-5)
Supplement: Supplementary file 1 — Supplementary Material 1 [file 12872_2023_3638_MOESM1_ESM.docx]

**Table S6.** Multivariable Cox Regression Analyses for Mortality after Excluding Participants with History of Cancer

|  | lnSII | | | | | Per SD increment in lnSII |
| --- | --- | --- | --- | --- | --- | --- |
|  | ≤5.84 | 5.84-6.18 | 6.18-6.54 | >6.54 | *P*_trend_ |  |
| All-cause mortality |  |  |  |  |  |  |
| Death, No./total No. | 407/1875 | 440/1868 | 490/1868 | 640/1874 |  |  |
| Model 1 | Reference | 1.00(0.84,1.19) | 0.98(0.80,1.20) | 1.45(1.25,1.68) | <0.001 | 1.22(1.15,1.30) |
| Model 2 | Reference | 0.96(0.79,1.16) | 0.99(0.79,1.24) | 1.40(1.16,1.68) | <0.001 | 1.21(1.12,1.30) |
| Model 3 | Reference | 1.00(0.81,1.24) | 0.97(0.78,1.21) | 1.36(1.10,1.67) | 0.003 | 1.18(1.08,1.28) |
| CVD mortality |  |  |  |  |  |  |
| Death, No. | 149 | 159 | 182 | 213 |  |  |
| Model 1 | Reference | 1.03(0.80,1.32) | 0.98(0.73,1.33) | 1.54(1.13,2.09) | 0.01 | 1.32(1.17,1.49) |
| Model 2 | Reference | 0.92(0.69,1.22) | 1.00(0.72,1.39) | 1.51(1.04,2.21) | 0.03 | 1.37(1.17,1.59) |
| Model 3 | Reference | 1.01(0.74,1.37) | 1.03(0.72,1.47) | 1.53(1.00,2.33) | 0.05 | 1.35(1.13,1.61) |

**Notes:** Model 1: adjusted for age (continuous), sex (male or female) and ethnicity (non-Hispanic white, non-Hispanic black, Mexican American, or other); Model 2: further adjusted for BMI (<25, 25-30, ≥30 kg/m^2^), education level (less than high school, high school or equivalent, or college or above), family income-poverty ratio (0-1.0, 1.0-3.0, or >3.0), smoking status (never smoker, current smoker, or former smoker), drinking status (non-drinker, low-to-moderate drinker, heavy drinker, or former drinker); Model 3: further adjusted for duration of diabetes (≤3, 3-10, or >10 years), diabetic medication use (none, only oral medication, insulin, or others), HbA1c (<7%, or ≥7%), hypertension, hyperlipidemia, CKD (yes, or no).
