# Supplementary material for: Association of systemic immune inflammatory index with all-cause and cause-specific mortality among individuals with type 2 diabetes
Source: BMC Cardiovasc Disord. 2023 Dec 6;23:596. doi: 10.1186/s12872-023-03638-5 (PMC10702126; doi:10.1186/s12872-023-03638-5)
Supplement: Supplementary file 2 — Supplementary Material 2 [file 12872_2023_3638_MOESM2_ESM.docx]

**Table S2.** Univariate Analysis of COX Regression Model

|  | HR (95% CI) | P-value |
| --- | --- | --- |
| Age | 1.08(1.07,1.09) | <0.001 |
| BMI | 0.97(0.96,0.98) | <0.001 |
| Poverty | 0.83(0.80,0.86) | <0.001 |
| lnSII | 1.36(1.21,1.52) | <0.001 |
| FBG | 1.02(1.00,1.03) | 0.08 |
| HOMA-IR | 1.01(1.00,1.01) | <0.001 |
| HbA1c | 0.98(0.95,1.01) | 0.11 |
| TG | 0.99(0.95,1.03) | 0.50 |
| TC | 0.92(0.88,0.97) | 0.002 |
| HDL | 1.36(1.12,1.64) | 0.002 |
| LDL | 0.84(0.76,0.93) | <0.001 |
| Gender |  |  |
| Male | Reference |  |
| Female | 0.91(0.81,1.01) | 0.08 |
| Ethnicity |  |  |
| Non-Hispanic White | Reference |  |
| Non-Hispanic Black | 0.77(0.68,0.87) | <0.001 |
| Mexican American | 0.48(0.40,0.57) | <0.001 |
| Other | 0.53(0.44,0.64) | <0.001 |
| Education levels |  |  |
| Less than high school | Reference |  |
| High school or equivalent | 0.75(0.66,0.85) | <0.001 |
| College or above | 0.59(0.52,0.67) | <0.001 |
| Smoking status |  |  |
| Never | Reference |  |
| Current | 1.30(1.12,1.52) | <0.001 |
| Former | 1.68(1.47,1.92) | <0.001 |
| Drinking status |  |  |
| Never | Reference |  |
| Mild-to-moderate | 0.73(0.60,0.87) | <0.001 |
| Heavy | 0.50(0.40,0.63) | <0.001 |
| Former | 1.29(1.11,1.51) | 0.001 |
| Hypertension |  |  |
| No | Reference |  |
| Yes | 2.10(1.84,2.40) | <0.001 |
| Hyperlipidemia |  |  |
| No | Reference |  |
| Yes | 1.36(1.21,1.52) | <0.001 |
| ASCVD |  |  |
| No | Reference |  |
| Yes | 2.84(2.53,3.18) | <0.001 |
| CKD |  |  |
| No | Reference |  |
| Yes | 3.28(2.88,3.74) | <0.001 |
