# Supplementary material for: Association of systemic immune inflammatory index with all-cause and cause-specific mortality among individuals with type 2 diabetes
Source: BMC Cardiovasc Disord. 2023 Dec 6;23:596. doi: 10.1186/s12872-023-03638-5 (PMC10702126; doi:10.1186/s12872-023-03638-5)
Supplement: Supplementary file 3 — Supplementary Material 3 [file 12872_2023_3638_MOESM3_ESM.docx]

**Table S3.** Stratified Analyses of the Associations between lnSII and All-cause Mortality among Diabetes

|  | lnSII | | | | | *P* interaction |
| --- | --- | --- | --- | --- | --- | --- |
|  | ≤5.84 | 5.84-6.19 | 6.19-6.55 | >6.55 | *P* trend |  |
| Age |  |  |  |  |  | 0.41 |
| ≤60 years | Reference | 1.12(0.78,1.62) | 0.84(0.55,1.27) | 1.31(0.92,1.86) | 0.24 |  |
| >60 years | Reference | 0.95(0.76,1.19) | 0.99(0.80,1.23) | 1.40(1.12,1.75) | <0.001 |  |
| Sex |  |  |  |  |  | 0.15 |
| Male | Reference | 1.00(0.76,1.32) | 0.91(0.70,1.19) | 1.50(1.15,1.95) | 0.002 |  |
| Female | Reference | 0.87(0.68,1.12) | 0.89(0.70,1.12) | 1.13(0.88,1.44) | 0.2 |  |
| Ethnicity |  |  |  |  |  | 0.18 |
| Non-Hispanic White | Reference | 0.87(0.66,1.14) | 0.82(0.63,1.07) | 1.20(0.94,1.53) | 0.05 |  |
| Other | Reference | 1.09(0.87,1.36) | 1.06(0.86,1.32) | 1.64(1.29,2.08) | <0.001 |  |
| Smoking status |  |  |  |  |  | 0.29 |
| Nonsmoker | Reference | 0.90(0.72,1.12) | 0.85(0.69,1.06) | 1.30(1.07,1.57) | 0.004 |  |
| Current smoker | Reference | 1.44(0.98,2.12) | 1.27(0.82,1.95) | 1.71(1.08,2.71) | 0.05 |  |
| BMI, kg/m^2^ |  |  |  |  |  | 0.33 |
| <30 | Reference | 0.93(0.72,1.21) | 1.00(0.79,1.27) | 1.40(1.09,1.79) | 0.004 |  |
| ≥30 | Reference | 0.91(0.71,1.18) | 0.78(0.61,1.01) | 1.23(0.95,1.60) | 0.11 |  |
| Duration of diabetes |  |  |  |  |  | 0.99 |
| ≤10 years | Reference | 0.93(0.75,1.16) | 0.89(0.72,1.11) | 1.30(1.04,1.63) | 0.02 |  |
| >10 years | Reference | 0.94(0.69,1.29) | 0.95(0.66,1.37) | 1.38(0.98,1.95) | 0.04 |  |
| Hypertension |  |  |  |  |  | 0.86 |
| No | Reference | 0.97(0.67,1.41) | 0.84(0.60,1.19) | 1.18(0.79,1.75) | 0.50 |  |
| Yes | Reference | 0.97(0.77,1.21) | 0.93(0.74,1.16) | 1.37(1.11,1.68) | 0.001 |  |
| Hyperlipidemia |  |  |  |  |  | 0.03 |
| No | Reference | 1.51(0.89,2.57) | 1.21(0.67,2.18) | 1.80(1.06,3.05) | 0.04 |  |
| Yes | Reference | 0.88(0.73,1.07) | 0.85(0.70,1.03) | 1.24(1.02,1.50) | 0.02 |  |
| CKD |  |  |  |  |  | 0.06 |
| No | Reference | 0.89(0.68,1.16) | 0.73(0.54,0.99) | 1.20(0.95,1.51) | 0.18 |  |
| Yes | Reference | 0.98(0.76,1.27) | 1.04(0.84,1.30) | 1.40(1.10,1.79) | 0.003 |  |

**Notes:** adjusted for age, sex, ethnicity, BMI, education level, family income-poverty ratio, smoking status, drinking status, duration of diabetes, diabetic medication use, HbA1c levels, hypertension, hyperlipidemia, ASCVD, CKD.
