# Supplementary material for: Association of systemic immune inflammatory index with all-cause and cause-specific mortality among individuals with type 2 diabetes
Source: BMC Cardiovasc Disord. 2023 Dec 6;23:596. doi: 10.1186/s12872-023-03638-5 (PMC10702126; doi:10.1186/s12872-023-03638-5)
Supplement: Supplementary file 4 — Supplementary Material 4 [file 12872_2023_3638_MOESM4_ESM.docx]

**Table S5.** Multivariable Cox Regression Analyses for Mortality after Excluding Participants with History of ASCVD

|  | lnSII | | | | | Per SD increment in lnSII |
| --- | --- | --- | --- | --- | --- | --- |
|  | ≤5.84 | 5.84-6.17 | 6.17-6.54 | >6.54 | *P*_trend_ |  |
| All-cause mortality |  |  |  |  |  |  |
| Death, No./total No. | 319/1664 | 345/1665 | 375/1663 | 485/1664 |  |  |
| Model 1 | Reference | 0.98(0.81,1.19) | 0.93(0.74,1.17) | 1.40(1.16,1.69) | <0.001 | 1.20(1.11,1.30) |
| Model 2 | Reference | 0.91(0.74,1.13) | 0.94(0.74,1.20) | 1.41(1.13,1.76) | <0.001 | 1.21(1.10,1.32) |
| Model 3 | Reference | 0.90(0.71,1.13) | 0.85(0.67,1.08) | 1.28(1.00,1.63) | 0.02 | 1.15(1.04,1.27) |
| CVD mortality |  |  |  |  |  |  |
| Death, No. | 85 | 106 | 131 | 147 |  |  |
| Model 1 | Reference | 1.28(0.93,1.76) | 1.21(0.84,1.76) | 2.07(1.38,3.12) | 0.001 | 1.49(1.29,1.72) |
| Model 2 | Reference | 1.10(0.77,1.56) | 1.23(0.83,1.82) | 2.13(1.37,3.30) | <0.001 | 1.57(1.34,1.84) |
| Model 3 | Reference | 1.08(0.73,1.60) | 1.11(0.73,1.69) | 1.88(1.16,3.06) | 0.01 | 1.48(1.23,1.79) |

**Notes:** Model 1: adjusted for age (continuous), sex (male or female) and ethnicity (non-Hispanic white, non-Hispanic black, Mexican American, or other); Model 2: further adjusted for BMI (<25, 25-30, ≥30 kg/m^2^), education level (less than high school, high school or equivalent, or college or above), family income-poverty ratio (0-1.0, 1.0-3.0, or >3.0), smoking status (never smoker, current smoker, or former smoker), drinking status (non-drinker, low-to-moderate drinker, heavy drinker, or former drinker); Model 3: further adjusted for duration of diabetes (≤3, 3-10, or >10 years), diabetic medication use (none, only oral medication, insulin, or others), HbA1c (<7%, or ≥7%), hypertension, hyperlipidemia, CKD (yes, or no).
