# Supplementary material for: Association of systemic immune inflammatory index with all-cause and cause-specific mortality among individuals with type 2 diabetes
Source: BMC Cardiovasc Disord. 2023 Dec 6;23:596. doi: 10.1186/s12872-023-03638-5 (PMC10702126; doi:10.1186/s12872-023-03638-5)
Supplement: Supplementary file 5 — Supplementary Material 5 [file 12872_2023_3638_MOESM5_ESM.docx]

**Table S1.** Baseline Characteristics of Participants with Diabetes according to All-cause Mortality

|  | Alive | Death | P-value |
| --- | --- | --- | --- |
| Participants, No. | 6205(71.59) | 2463(28.41) |  |
| Age, mean (SE), y | 56.26(0.26) | 68.15(0.36) | <0.001 |
| Gender |  |  | 0.22 |
| Male | 3067(50.41) | 1392(52.47) |  |
| Female | 3138(49.59) | 1071(47.53) |  |
| Ethnicity |  |  | <0.001 |
| Non-Hispanic White | 1931(59.08) | 1244(72.41) |  |
| Non-Hispanic Black | 1531(14.40) | 582(13.35) |  |
| Mexican American | 1350(10.53) | 409( 5.22) |  |
| Other | 1393(15.98) | 228( 9.01) |  |
| BMI |  |  | <0.001 |
| <25.0 | 756(10.70) | 444(18.10) |  |
| 25.0-29.9 | 1776(26.57) | 778(30.47) |  |
| ≥30 | 3575(62.73) | 1080(51.43) |  |
| HEI, mean (SE) | 50.83(0.27) | 52.18(0.36) | 0.003 |
| Smoking status |  |  | <0.001 |
| Never | 3301(52.39) | 1019(40.19) |  |
| Current | 997(16.23) | 409(17.94) |  |
| Former | 1887(31.38) | 1033(41.87) |  |
| Drinking status |  |  | <0.001 |
| Never | 1006(15.34) | 452(18.96) |  |
| Mild-to-moderate | 1813(37.52) | 561(27.51) |  |
| Heavy | 1400(27.61) | 308(14.14) |  |
| Former | 1242(19.53) | 928(39.39) |  |
| Education levels |  |  | <0.001 |
| Less than high school | 2076(21.78) | 1155(36.43) |  |
| High school or equivalent | 1416(25.44) | 560(26.06) |  |
| College or above | 2705(52.78) | 740(37.51) |  |
| Family income-poverty ratio |  |  | <0.001 |
| ≤1.0 | 1320(15.93) | 548(19.50) |  |
| 1.0-3.0 | 2439(38.71) | 1192(51.60) |  |
| >3.0 | 1828(45.36) | 490(28.90) |  |
| Duration of diabetes |  |  | <0.001 |
| ≤3 years | 3157(52.60) | 1022(42.61) |  |
| 3-10 years | 1425(22.66) | 498(21.15) |  |
| >10 years | 1623(24.74) | 943(36.24) |  |
| FBG, mmol/L | 8.36(0.07) | 8.68(0.13) | 0.02 |
| HOMA-IR | 8.40(0.25) | 9.35(0.49) | 0.08 |
| HbA1c, % |  |  | 0.59 |
| <7.0 | 3515(59.50) | 1391(58.56) |  |
| ≥7.0 | 2675(40.50) | 1062(41.44) |  |
| Diabetes medication use |  |  | <0.001 |
| No insulin or pills | 2423(40.43) | 843(35.10) |  |
| Only diabetes pills | 2728(42.56) | 1013(39.84) |  |
| Only insulin | 438( 7.50) | 361(15.55) |  |
| Pills and insulin | 616(9.52) | 246(9.51) |  |
| Self-reported disease |  |  |  |
| Hypertension | 4234(65.75) | 1974(80.34) | <0.001 |
| Hyperlipidemia | 5353(87.69) | 2126(87.64) | 0.96 |
| ASCVD | 1073(16.73) | 939(38.00) | <0.001 |
| CKD | 1997(29.78) | 1506(60.82) | <0.001 |
| TG, mmol/L | 1.89(0.04) | 2.02(0.05) | 0.05 |
| TC, mmol/L | 4.88(0.02) | 4.93(0.03) | 0.21 |
| HDL, mmol/L | 1.22(0.01) | 1.26(0.01) | 0.01 |
| LDL, mmol/L | 2.79(0.03) | 2.72(0.04) | 0.13 |

**Notes:** Data are numbers (percentages) unless otherwise indicated. All estimates accounted for complex survey designs, and all percentages were weighted.

**Abbreviations:** BMI, body mass index (calculated as weight in kilograms divided by height in meters squared); HEI, Healthy Eating Index; MET, metabolic equivalent; HbA1c, glycated hemoglobin A1c; NHANES, National Health an Nutrition Examination Survey.
