# Supplementary material for: Association of systemic immune inflammatory index with all-cause and cause-specific mortality among individuals with type 2 diabetes
Source: BMC Cardiovasc Disord. 2023 Dec 6;23:596. doi: 10.1186/s12872-023-03638-5 (PMC10702126; doi:10.1186/s12872-023-03638-5)
Supplement: Supplementary file 6 — Supplementary Material 6 [file 12872_2023_3638_MOESM6_ESM.docx]

**Table S8.** Multivariable Cox Regression Analyses for Mortality with Further Adjustment of HEI, HOMA-IR, and Blood Lipid

|  | lnSII | | | | | Per SD increment in lnSII |
| --- | --- | --- | --- | --- | --- | --- |
|  | ≤5.84 | 5.84-6.19 | 6.19-6.55 | >6.55 | *P*_trend_ |  |
| All-cause mortality |  |  |  |  |  |  |
| Model 1 | Reference | 0.99(0.85,1.16) | 0.97(0.81,1.15) | 1.44(1.25,1.65) | <0.001 | 1.21(1.14,1.28) |
| Model 2 | Reference | 0.95(0.79,1.16) | 0.92(0.75,1.12) | 1.33(1.10,1.61) | 0.002 | 1.17(1.07,1.26) |
| Model 2 + HEI | Reference | 0.95(0.78,1.14) | 0.92(0.75,1.12) | 1.32(1.09,1.59) | 0.002 | 1.16(1.07,1.26) |
| Model 2 + HOMA-IR | Reference | 0.85(0.66,1.10) | 0.94(0.74,1.20) | 1.24(0.96,1.60) | 0.03 | 1.16(1.04,1.28) |
| Model 2 + TG + HDL + LDL | Reference | 0.78(0.59,1.02) | 0.89(0.69,1.15) | 1.18(0.91,1.55) | 0.05 | 1.16(1.04,1.28) |
| CVD mortality |  |  |  |  |  |  |
| Model 1 | Reference | 1.10(0.89,1.37) | 1.03(0.77,1.37) | 1.65(1.25,2.18) | <0.001 | 1.33(1.19,1.50) |
| Model 2 | Reference | 1.03(0.79,1.34) | 1.07(0.77,1.50) | 1.59(1.11,2.27) | 0.01 | 1.34(1.15,1.57) |
| Model 2 + HEI | Reference | 1.02(0.78,1.33) | 1.08(0.77,1.53) | 1.59(1.10,2.28) | 0.01 | 1.34(1.14,1.57) |
| Model 2 + HOMA-IR | Reference | 0.94(0.64,1.39) | 1.13(0.75,1.69) | 1.60(1.04,2.47) | 0.02 | 1.28(1.07,1.53) |
| Model 2 + TG + HDL + LDL | Reference | 0.93(0.62,1.40) | 1.08(0.71,1.65) | 1.63(1.01,2.63) | 0.02 | 1.31(1.08,1.59) |

**Notes:** Model 1: adjusted for age (continuous), sex (male or female) and ethnicity (non-Hispanic white, non-Hispanic black, Mexican American, or other);

Model 2: further adjusted for BMI (<25, 25-30, ≥30 kg/m^2^), education level (less than high school, high school or equivalent, or college or above), family income-poverty ratio (0-1.0, 1.0-3.0, or >3.0), smoking status (never smoker, current smoker, or former smoker), drinking status (non-drinker, low-to-moderate drinker, heavy drinker, or former drinker), duration of diabetes (≤3, 3-10, or >10 years), diabetic medication use (none, only oral medication, insulin, or others), HbA1c (<7%, or ≥7%), hypertension, hyperlipidemia, ASCVD, CKD (yes, or no).
