# Supplementary material for: Association of systemic immune inflammatory index with all-cause and cause-specific mortality among individuals with type 2 diabetes
Source: BMC Cardiovasc Disord. 2023 Dec 6;23:596. doi: 10.1186/s12872-023-03638-5 (PMC10702126; doi:10.1186/s12872-023-03638-5)
Supplement: Supplementary file 7 — Supplementary Material 7 [file 12872_2023_3638_MOESM7_ESM.docx]

**Table S4.** Stratified Analyses of the Associations between lnSII and CVD Mortality among Diabetes

|  | lnSII | | | | | *P* interaction |
| --- | --- | --- | --- | --- | --- | --- |
|  | ≤5.84 | 5.84-6.19 | 6.19-6.55 | >6.55 | *P* trend |  |
| Age |  |  |  |  |  | 0.02 |
| ≤60 years | Reference | 1.95(1.10,3.47) | 1.06(0.55,2.05) | 1.66(0.80,3.44) | 0.49 |  |
| >60 years | Reference | 0.87(0.63,1.21) | 1.10(0.76,1.60) | 1.59(1.08,2.33) | 0.01 |  |
| Sex |  |  |  |  |  | 0.01 |
| Male | Reference | 1.05(0.71,1.55) | 1.20(0.76,1.89) | 2.06(1.29,3.29) | 0.002 |  |
| Female | Reference | 0.91(0.60,1.37) | 0.90(0.62,1.31) | 1.05(0.68,1.63) | 0.75 |  |
| Ethnicity |  |  |  |  |  | 0.52 |
| Non-Hispanic White | Reference | 0.93(0.64,1.35) | 0.94(0.61,1.45) | 1.37(0.86,2.19) | 0.11 |  |
| Other | Reference | 1.09(0.69,1.70) | 1.38(0.93,2.04) | 1.89(1.26,2.82) | 0.001 |  |
| Smoking status |  |  |  |  |  | 0.05 |
| Nonsmoker | Reference | 0.88(0.65,1.20) | 1.00(0.71,1.40) | 1.52(1.06,2.16) | 0.01 |  |
| Current smoker | Reference | 2.92(1.39,6.13) | 2.26(0.87,5.87) | 2.46(0.91,6.64) | 0.23 |  |
| BMI, kg/m^2^ |  |  |  |  |  | 0.33 |
| <30 | Reference | 0.94(0.61,1.46) | 1.18(0.76,1.84) | 1.45(0.92,2.29) | 0.08 |  |
| ≥30 | Reference | 0.95(0.66,1.37) | 0.87(0.57,1.32) | 1.50(1.00,2.24) | 0.04 |  |
| Duration of diabetes |  |  |  |  |  | 0.90 |
| ≤10 years | Reference | 1.04(0.73,1.47) | 1.05(0.72,1.54) | 1.56(1.04,2.33) | 0.02 |  |
| >10 years | Reference | 0.87(0.54,1.41) | 1.05(0.60,1.84) | 1.41(0.82,2.44) | 0.12 |  |
| Hypertension |  |  |  |  |  | 0.27 |
| No | Reference | 0.74(0.35,1.55) | 0.97(0.46,2.02) | 1.48(0.77,2.87) | 0.2 |  |
| Yes | Reference | 1.13(0.83,1.54) | 1.11(0.77,1.59) | 1.57(1.07,2.31) | 0.02 |  |
| Hyperlipidemia |  |  |  |  |  | 0.001 |
| No | Reference | 5.16(1.35,19.71) | 5.88(1.40,24.63) | 10.20(2.91,35.70) | <0.001 |  |
| Yes | Reference | 0.89(0.67,1.18) | 0.95(0.68,1.32) | 1.29(0.89,1.87) | 0.12 |  |
| CKD |  |  |  |  |  | 0.09 |
| No | Reference | 0.91(0.56,1.46) | 0.70(0.40,1.23) | 1.20(0.74,1.94) | 0.52 |  |
| Yes | Reference | 0.97(0.68,1.38) | 1.24(0.88,1.75) | 1.57(1.03,2.39) | 0.02 |  |

**Notes:** Adjusted for age, sex, ethnicity, BMI, education level, family income-poverty ratio, smoking status, drinking status, duration of diabetes, diabetic medication use, HbA1c levels, hypertension, hyperlipidemia, ASCVD, CKD.
