# Supplementary material for: Association of systemic immune inflammatory index with all-cause and cause-specific mortality among individuals with type 2 diabetes
Source: BMC Cardiovasc Disord. 2023 Dec 6;23:596. doi: 10.1186/s12872-023-03638-5 (PMC10702126; doi:10.1186/s12872-023-03638-5)
Supplement: Supplementary file 8 — Supplementary Material 8 [file 12872_2023_3638_MOESM8_ESM.docx]

**Table S9.** Missing of variables

| Variables | Missing (n) | Missing proportion (%) |
| --- | --- | --- |
| Family income-poverty ratio | 851 | 9.82 |
| Education levels | 16 | 0.18 |
| BMI | 259 | 2.99 |
| Hypertension | 2 | 0.02 |
| Hyperlipidemia | 1 | 0.01 |
| CKD | 275 | 3.17 |
| ASCVD | 23 | 0.27 |
| Cancer | 36 | 0.42 |
| Smoking status | 22 | 0.25 |
| Drinking status | 958 | 11.05 |
| HEI | 601 | 6.93 |
| HbA1c | 25 | 0.29 |
