# Supplementary material for: Association of systemic immune inflammatory index with all-cause and cause-specific mortality among individuals with type 2 diabetes
Source: BMC Cardiovasc Disord. 2023 Dec 6;23:596. doi: 10.1186/s12872-023-03638-5 (PMC10702126; doi:10.1186/s12872-023-03638-5)
Supplement: Supplementary file 9 — Supplementary Material 9 [file 12872_2023_3638_MOESM9_ESM.docx]

**Table S7.** Multivariable Cox Regression Analyses for Mortality after Excluding Participants who Died within 2 years

|  | lnSII | | | | | Per SD increment in lnSII |
| --- | --- | --- | --- | --- | --- | --- |
|  | ≤5.84 | 5.84-6.18 | 6.18-6.54 | >6.54 | *P*_trend_ |  |
| All-cause mortality |  |  |  |  |  |  |
| Death, No./total No. | 424/2064 | 470/2066 | 514/2064 | 647/2066 |  |  |
| Model 1 | Reference | 1.01(0.85,1.19) | 0.94(0.77,1.14) | 1.35(1.16,1.58) | <0.001 | 1.17(1.10,1.25) |
| Model 2 | Reference | 0.96(0.79,1.16) | 0.95(0.76,1.19) | 1.34(1.10,1.63) | 0.002 | 1.16(1.08,1.26) |
| Model 3 | Reference | 0.98(0.79,1.21) | 0.92(0.74,1.15) | 1.26(1.02,1.57) | 0.03 | 1.13(1.03,1.23) |
| CVD mortality |  |  |  |  |  |  |
| Death, No. | 143 | 164 | 187 | 216 |  |  |
| Model 1 | Reference | 1.17(0.91,1.50) | 1.07(0.79,1.44) | 1.67(1.23,2.25) | 0.003 | 1.30(1.15,1.47) |
| Model 2 | Reference | 1.04(0.78,1.40) | 1.10(0.80,1.52) | 1.63(1.16,2.30) | 0.005 | 1.32(1.15,1.52) |
| Model 3 | Reference | 1.12(0.82,1.53) | 1.15(0.81,1.63) | 1.59(1.09,2.33) | 0.02 | 1.28(1.10,1.49) |

Notes: Model 1: adjusted for age (continuous), sex (male or female) and ethnicity (non-Hispanic white, non-Hispanic black, Mexican American, or other); Model 2: further adjusted for BMI (<25, 25-30, ≥30 kg/m^2^), education level (less than high school, high school or equivalent, or college or above), family income-poverty ratio (0-1.0, 1.0-3.0, or >3.0), smoking status (never smoker, current smoker, or former smoker), drinking status (non-drinker, low-to-moderate drinker, heavy drinker, or former drinker), MET (continuous); Model 3: further adjusted for duration of diabetes (≤3, 3-10, or >10 years), diabetic medication use (none, only oral medication, insulin, or others), HbA1c (<7%, or ≥7%), hypertension, hyperlipidemia, ASCVD, CKD (yes, or no).
