# Supplementary material for: Association of systemic immune inflammatory index with all-cause and cause-specific mortality among individuals with type 2 diabetes
Source: BMC Cardiovasc Disord. 2023 Dec 6;23:596. doi: 10.1186/s12872-023-03638-5 (PMC10702126; doi:10.1186/s12872-023-03638-5)
Supplement: Supplementary file 10 — Supplementary Material 10 [file 12872_2023_3638_MOESM10_ESM.docx]

**Supplementary Figure Legends**

Supplementary Figure 1. Restricted cubic spline regression for the associations between lnSII and cancer mortality.

Supplementary Figure 2. Restricted cubic spline regression for the associations between lnSII and CVD mortality in different subgroups. Male (A) and female (B). Without hyperlipidemia (C) or with hyperlipidemia (D).

Supplementary Figure 3. Results for multiple imputations (5 times) of missing data. All-cause mortality (A) and CVD mortality (B).
